# Molecular Detection of Invasive Species in Heterogeneous Mixtures Using a Microfluidic Carbon Nanotube Platform

**DOI:** 10.1371/journal.pone.0017280

**Published:** 2011-02-18

**Authors:** Andrew R. Mahon, Matthew A. Barnes, Satyajyoti Senapati, Jeffrey L. Feder, John A. Darling, Hsueh-Chia Chang, David M. Lodge

**Affiliations:** 1 Department of Biological Sciences, Center for Aquatic Conservation, The University of Notre Dame, Notre Dame, Indiana, United States of America; 2 Department of Chemical and Biomolecular Engineering, The University of Notre Dame, Notre Dame, Indiana, United States of America; 3 National Exposure Research Laboratory, Molecular Ecology Research Branch, United States Environmental Protection Agency, Cincinnati, Ohio, United States of America; University of Houston, United States of America

## Abstract

Screening methods to prevent introductions of invasive species are critical for the protection of environmental and economic benefits provided by native species and uninvaded ecosystems. Coastal ecosystems worldwide remain vulnerable to damage from aquatic species introductions, particularly via ballast water discharge from ships. Because current ballast management practices are not completely effective, rapid and sensitive screening methods are needed for on-site testing of ships in transit. Here, we describe a detection technology based on a microfluidic chip containing DNA oligonucleotide functionalized carbon nanotubes. We demonstrate the efficacy of the chip using three ballast-transported species either established (*Dreissena bugensis*) or of potential threat (*Eriocheir sinensis* and *Limnoperna fortuneii*) to the Laurentian Great Lakes. With further refinement for on-board application, the technology could lead to real-time ballast water screening to improve ship-specific management and control decisions.

## Introduction

Since European settlement of North America, over 180 aquatic invasive species have become established in the Great Lakes, many causing severe environmental and economic damage [1], [2]. During recent decades, the majority of aquatic invasive species (∼70%) entered the Great Lakes through transoceanic shipping routes, most through the ballast water of incoming ships [3]–[6]. Damages caused by these organisms can be substantial; over 40% of the invaders have been classified as harmful, and some, like the zebra mussel *Dreissena polymorpha*, have caused considerable ecosystem harm to the Great Lakes [7]. In addition to biological costs, non-indigenous species have had great economic impacts. For example, estimated costs of the zebra mussel invasion that began in the Great Lakes and now extends across much of the contiguous US amount to over $100 billion annually [8], [9].

The high costs of aquatic invasive species call for more effective preventative measures [9]. Unfortunately, the most commonly used ballast management strategy, ballast water exchange, is of unknown effectiveness [10], and ballast water treatment technologies are mostly still in the research and development phase [11]. An urgent need therefore exists for rapid detection methods to identify harmful invasive species in ships' ballast prior to port entry to provide adequate time for taking appropriate control actions. Even if ballast water treatment systems become widely adopted, the need will exist for rapid inspection and enforcement tools. Traditional microscope-based methods have been used to examine ballast samples for target organisms, but are expensive and slow [12]. By the time results from microscopic examination are available, ships have long since reached their destinations, making it too late to intervene [6]. In addition, larvae, which are the most often encountered life stage in ballast tanks, are often not identifiable to the species level using morphological keys [22]. Accordingly, a rapid and sensitive on-board detection platform that can identify target species in large volume ballast samples requiring minimal user expertise would greatly facilitate voluntary or regulatory inspection, enforcement, and control efforts.

**Figure 1:**
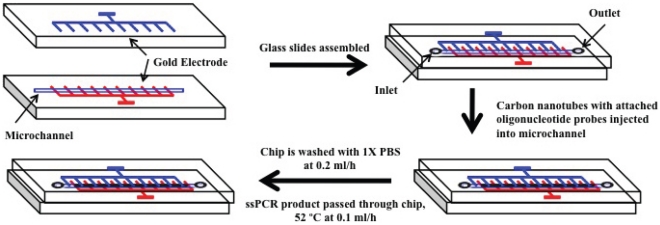
Schematic diagram of carbon nanotube chip design and its functionality. Chip design, fabrication, and processing are described elsewhere [13].

Here, we test a novel method for ballast water screening involving a microfluidic detection platform. The system couples standard polymerase chain reaction (PCR) techniques (including DNA extraction from whole ballast water samples and amplification of species-specific sequences) with an open-flow, carbon nanotube microfluidic detection chip to identify target organisms of concern (Fig. 1). The system detects changes in impedance caused by the hybridization of DNA from targeted invasive taxa to species-specific oligonucleotide probes attached to carbon nanotubes. The system is inexpensive to manufacture, and because it does not require fluorescence or radio- labels or manipulation of PCR amplification products, has potential for development into a single chip platform for field application.

## Results

To initiate detection, total DNA is first isolated from a water sample and asymmetric PCR performed to generate single stranded DNA for the mitochondrial *cytochrome c oxidase subunit I* (COI) gene of a target species of concern. Although we chose COI here for demonstration purposes, any region possessing a species-specific diagnostic sequence could be used. Amplified DNA was then introduced into the detection chip's microchannel (Fig. 1), which contained carbon nanotubes functionalized with 26 base-pair long DNA oligonucleotides reverse complement to the PCR amplified, species diagnostic COI sequence. An alternating current (AC) field held the functionalized carbon nanotubes in the microchannel (Fig. 1). When amplified COI products from the target species bind to the species-diagnostic oligonucleotide probes attached to the carbon nanotubes, impedance values increase in the AC field. Non-target DNA molecules do not bind to the probes and pass through the system. Preliminary trials indicated that such a DNA:DNA hybridization-based methodology can rapidly and accurately discriminate between closely related congeneric species differing by only three nucleotides over a 26 base targeted COI binding region [13].

To test the efficacy of the detection chip, we performed a series of experiments on lab-constructed ballast water samples containing 1) DNA from only a single target species of interest at concentrations as low as a single larval equivalent per sample, 2) complex mixtures of multiple species that included a large excess of non-target organism DNA, and 3) several different types of negative controls, some blank and others including DNA from target species different from the probes attached to the functionalized carbon nanotubes. Our target organisms were *Dreissena bugensis* (quagga mussel), a species that is known to be in the Great Lakes with viable populations; *Eriocheir sinensis* (Chinese mitten crab), a species that has been reported in the Great Lakes, is likely to be able to establish there [14], but is thought not yet to be reproducing [15]; and *Limnoperna fortunei* (golden mussel), a native to central and southeastern Asia, which has invaded South America's Rio de La Plata, Rio Paraná, Rio Uruguay, and Rio Paraguay [16], and is likely to find the Great Lakes suitable habitat if introduced [17]. Our non-target species was the cladoceran *Daphnia magna*.

**Figure 2:**
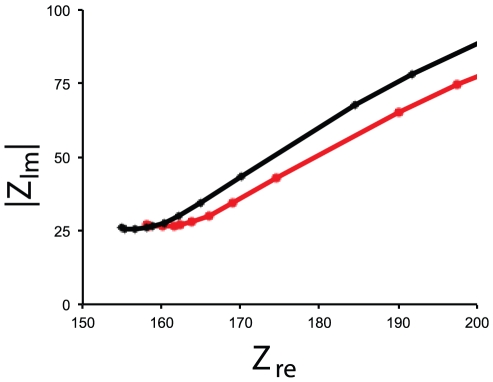
Nyquist plots (realized impedance change (Z_re_) vs. imaginary impedance change[**13**] (|Z_im_|) of impedance measurement from the ssPCR product of a homogenous DNA sample from *Dreissena bugensis* containing only target organism (black squares) genetic material compared to negative control (background; red triangles). Positive reactions on the CNT platform are visually identified by either an increase in slope or a shift to the left of the impedance curve relative to the negative control (background) curve. Tests on heterogeneous DNA samples were similarly successful. The heterogeneous samples (100 µL total volume) consisted of genomic DNA from the three target invasive species (*D. bugensis*, *L. fortunei*, and *E. sinensis*) and the non-target cladoceran *Daphnia magna* (Table 2).

For each target species, asymmetric, single strand PCR amplification was performed using species-specific COI primers (Table 1). The resulting PCR amplified samples were tested in a double-blind experiment on the carbon nanotube platform and compared to background signal levels generated from negative controls (Fig. 2). Results for pure species samples were positive for all three target organisms, while all negative controls, including non-target organism tests, produced negative results.

**Table 1:** HCO-2198 used as reverse primer in ssPCR amplification [20]; *D. bugensis* forward primer from C. Nowak, pers. com. *Limnoperna* markers from Pie et al. [16].

Target Species	Forward Primer	Internal Target Tag
*D. bugensis*	5′- CCTTATTATTCTGTTCGGCGTTTAG-3′	5′- TGTTCAACCCCCACCAAATCCGCCCTCA-3′
*E. sinensis*	5′- TGGCAACTGACTTGTCCCTCTTATACTAGG-3′	5′- AGGTGGGTAGACAGTCCACCCAGTACCAA-3′
*L. fortunei*	5′- TTTAGAGTTAGCACGTCCTGGTAGGTT-3′	5′- TCCAACCAGTCCCTACTCCACCCTCTA-3′

**Figure 3:**
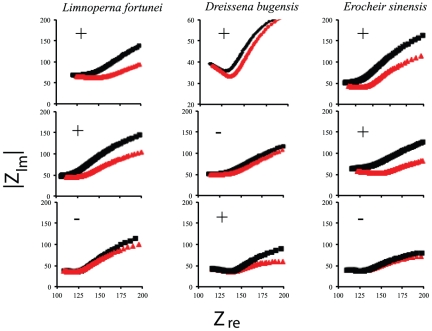
Nyquist plots (Realized impedance change (Z_re_) vs. imaginary impedance change [**13**] (|Z_im_|) for heterogeneous sample tests for *Limnoperna fortunei*, *Dreissena bugensis*, and *Erocheir sinensis.* Each column represents the tests of each heterogeneous sample for the identified target species (n = 3). Black squares indicate test samples and red triangles indicate background signal. Symbols on each individual chart indicate the result of the detection experiment for the target species. The accuracy of our detection in the double-blind tests was 100%.

We chose to utilize artificial ballast samples in this series of experiments to ensure the presence of target organisms. The heterogeneous species mixtures we created with target species and non-target cladocerans included more non-target organisms than would have been gathered in a large volume ballast sample (Mahon, unpublished data). Additionally, testing real ballast samples would have presented the experimental problem of not knowing the precise constituents of the samples (i.e. we would not have had a priori knowledge of the presence or absence of target species). Tests (n = 9) on heterogeneous DNA samples (100 µL total volume) containing mixtures of the three target invasive species (*D. bugensis*, *L. fortunei*, and *E. sinensis*) and the non-target cladoceran *Daphnia magna* (background DNA) were also 100% accurate (Fig. 3). Each of the three heterogeneous mixtures was tested for each target organism after a background reading was taken for the species-specific CNT microfluidic chip. Even with heterogeneous sample tests, the carbon nanotube platform detected the DNA equivalent of a single larva of target organisms in the presence of substantially higher (see Table 2) concentrations of non-target background DNA (Fig. 3).

**Table 2:** Concentrations of genomic DNA from target and non-target organisms present in heterogeneous samples.

	DNA amount in heterogeneous sample			
	*D. bugensis*	*L. fortunei*	*E. sinensis*	*Daphnia magna*
Target species: *Dreissena bugensis* (0.0285 µg/larvae)	---	1.53 µg	479.0 ng	7.54 µg
Target species: *Limnoperna fortunei* (0.0285 µg/larvae)	1.84 µg	---	479.0 ng	7.54 µg
Target species *Erocheir sinensis* (0.0723 µg/larvae)	1.84 µg	1.53 µg	---	7.54 µg

For each target organism, 100 µL of sample was prepared that included the equivalent amount of DNA from a single target organism larvae (see text), background DNA (*Daphnia magna*) and the other two non-target species at high concentrations. Additionally, for each target organism, blank samples that included DNA from non-target organisms and background DNA were also prepared and tested as the background for each heterogeneous sample.

## Discussion

To enable improved environmental protection, timely data are needed to determine if potentially invasive species are contained in particular ships entering the Great Lakes and other coastal regions of the world. Only then can the risks be understood at the spatial and temporal scales needed to make appropriate decisions to reduce the potential for harm. We believe that the further development and implementation of the technology tested here will provide vital information to guide decisions in the public and private sector, including those by government agencies, the shipping industry, insurers, and other stakeholders, as it will become possible to test rapidly for the presence of target organisms in specific ships. The tools described in this manuscript could enable regulators to ensure compliance in actionable time frames, and could be used by ship operators to document compliance with various enacted and proposed ballast water concentration standards [18]. Future directions and uses of this tool include processing large water samples, envisioned at volumes nearing 1000 L to gain insight as to the representative constituents of ballast tanks. Finally, ballast water monitoring is only one of many potential applications for the tested technology, including detection of invasive species in ports or other environments [19], threatened or endangered species, species of public health concern (e.g., pathogens, parasites, or indicator species), and species of concern for biosecurity. These other potential applications are frontiers for subsequent development and testing of this or similar technology.

## Methods

### Nanotube functionalization and sample preparation

Carboxilated MWNTs were commercially sourced (Cheaptubes.com; internal diameter <8 nm, length ∼10 nm). Functionalization of the multiwall carbon nanotubes (MWNT) with a species-specific oligonucleotide primer was performed as reported in the literature [13], [21]. The DNA markers (20–30 bases in length) are comprised of the complimentary DNA sequence to target species. Carboxilated nanotubes were first washed three times in 0.1 M 2-(N-morpholino) ethanesulfonic acid (MES) and spun at 2000 rpm for 1 minute (each run). The MWNTs were then resuspended in 0.1 M MES. Oligonucleotide primers were then attached to the carboxylated MWNTs using water-soluble 1-ethyl-3-(3-dimethylaminopropyl) carbodiimide (EDC) and *N*-hydroxysuccinimide (NHS) and slowly mixing for 30 min. This produced stable amide linkages binding the MWNTs and the oliginucleotude primers together [13], [21], [23]. Following this, the DNA-MWNTs were washed in 1X PBS three times and stored at 4°C until use in experiments. The EDC/NHS chemistry used to couple amine and carboxyl groups is very well established and the reactions themselves are noted in the literature as mild [13], [21].

The possibility of any side-product formation with amine groups present on the bases is negligible as the coupling reaction is thermodynamically/kinetically unfavorable due to excessive steric hindrance (spatial crowding). This provides little chance for isomer formation curing nanotube preparation. Since previous studies [13], [23] found that this procedure produced successful nanotube-oligonucleotide conjugates, further purification and characterization was not performed. Tam et al. further characterized this binding of DNA and MWNTs using FTIR and found the conjugation was reproducible and the resulting DNA-MWNTs were functional for further hybridization reactions [23]. Additionally, in the coupling reaction used to prepare our DNA-MWNTs, both reactants are achiral molecules and no chiral center is generated during the reaction. Thus, the question of isomer formation during coupling reaction is not applicable to this situation [13], [21].

Preparation of the carbon nanotube hybridization chip has been described previously [13]. Functionalized nanotubes are captured in the microchannel by application followed by applying an electric field (1 MHz and 1V) to the microelectrode chip (Figure 1) [13]. DNA extractions were performed on bulk *Daphnia magna* samples (∼1000 individuals; Carolina Biological Supply Company) using the PowerSoil DNA extraction kit (MoBio Inc.) per the manufacturer's recommendations. These organisms served as the background DNA for experiments. Final elutions of the DNA extractions were vacuum centrifuged from 5 mL to 1 mL total volume. Tissue extractions from target organisms were performed using Qiagen DNEasy Tissue extraction kit (Qiagen Inc.) per manufacturer's recommendations. DNA concentrations on all extractions were determined on a Nanodrop 2000 spectrophotometer (ThermoFisher Scientific).

### Polymerase Chain Reaction

Each PCR reaction consisted of 10X buffer (Eppendorf), 0.5 µL of dNTPs (10 mM stock), 3 µL Mg(OAc)_2_ (25 mM stock), 0.75 µL each primer (forward 10 mM stock, reverse 0.02 mM stock; Table 1), 0.132 µL Taq polymerase (Eppendorf; 5 U/µL), sterile water to 24 µL, and 1 µL DNA extract.

The PCR amplification thermal program to produce single stranded PCR product consisted of an initial denaturation at 94°C for 1 min, followed by 5 cycles of 94°C for 30 sec, 52°C for 30 sec., and 72°C for 1 min. This was then followed by 50 cycles of 94°C for 1 min, 52°C for 1 min, 72°C for 1∶30 and a final extension of 72°C for 5 min.

Protocols for hybridization of samples on the CNT chips and impedance measurement were described previously [13]. Flow rates for the samples through the CNT chip were 0.2 mL/h. This high flow rate provides a hydrodynamic shearing force that can cleave or alter the confirmation of any nonspecifically binding DNA fragments in the AC field.
